# The outcome of primary brachial plexus reconstruction in extended Erb’s obstetric palsy when two roots are available for intraplexus neurotization

**DOI:** 10.1007/s00238-016-1267-6

**Published:** 2017-01-06

**Authors:** Mohammad M. Al-Qattan, Amel A.F El-Sayed

**Affiliations:** 1 Department of Surgery, King Saud University, Riyadh, Saudi Arabia; 2 Department of Obstetrics and Gynecology, King Saud University, Riyadh, Saudi Arabia

**Keywords:** Obstetric brachial plexus, Erb’s palsy, Nerve graft

## Abstract

**Background:**

The outcome of primary brachial plexus reconstruction in extended Erb’s obstetric palsy with single root avulsion has not been specifically documented in the literature.

**Methods:**

A series of 46 consecutive cases of extended Erb’s obstetric palsy with single root avulsion was retrospectively reviewed. The upper and middle trunks were reconstructed with nerve grafts from the available two roots. No nerve transfers were used. The percentage of a satisfactory motor recovery was documented.

**Results:**

The postoperative motor recovery was excellent (over 97%) satisfactory outcome for elbow flexion, elbow extension, and digital extension. A satisfactory wrist extension was noted in 84.8% of children. The lowest rates of satisfactory outcomes were for shoulder external rotation (65.2%) and shoulder abduction (56.5%).

**Conclusions:**

In extended Erb’s obstetric palsy with single root avulsion, two ruptured roots are available for intraplexus neurotization of the upper and middle trunks. The surgeon gives a priority to elbow flexion and this is translated in an excellent outcome for elbow flexion. The triceps and digital extensors get a major contribution form the unaffected C8 root, and this is also translated in an excellent outcome for these two functions. Fewer cable grafts are available for reconstruction of the posterior division of upper trunk and the middle trunk, resulting in a lower rate of satisfactory outcomes at the shoulder for wrist extension.

Level of Evidence: Level IV, therapeutic study.

## Introduction

Although there are several studies [1–6] in the literature on the results of primary reconstruction of the brachial plexus in obstetric brachial plexus palsy (OBBP), the results of extended Erb’s obstetric palsy with single root avulsion has not been specifically investigated.

In extended Erb’s obstetric palsy with single root avulsion, the remaining two roots are available for intraplexus neurotization. The senior author’s reconstructive strategy in these cases has been the same over the last two decades of practice. In these cases, the upper and middle trunks (including the suprascapular nerve) are reconstructed with sural nerve cable grafts (utilizing the two available roots) without the use of any nerve transfers.

The main aim of the current article is to report on the results of a series of extended Erb’s obstetric palsy with single root avulsion treated only with intraplexus neurotization. A satisfactory outcome would indicate that nerve transfers are unnecessary in these cases, while an unsatisfactory outcome would suggest that the inclusion of nerve transfers should be tried in this group of infants.

## Material and methods

The study included all OBBP cases with extended Erb’s palsy (involving the C5, C6, and C7 roots) and single root avulsion and who were treated surgically by the senior author (between 1995 and 2013) by intraplexus sural nerve grafts before the age of 6 months. At our center, the indication for surgery in infants with extended Erb’s palsy is the lack of active elbow flexion against gravity by 3.5 months of age. However, many children present late, and delayed primary surgery may have an impact on the outcome of surgery. Hence, we decided to only include infants operated upon between 3.5 and 6 months. Children who were operated after 6 months of age and those who had a postoperative follow-up of less than 3 years were not included.

At our center, no MRI is done for infants with obstetric palsy to assess root avulsion. There are three main reasons for not including the MRI in the assessment of these infants. Firstly, MRI requires general anesthesia in infants. Secondly, MRI assessment of root avulsion is not 100% accurate. Finally, our indication for surgery is based only on the clinical assessment of active elbow flexion as mentioned above. Hence, the diagnosis of root avulsion in the current study is based on intraoperative assessment, with the visualization of the dorsal root ganglion outside the foramen.

The following data were collected at final follow-up prior to doing any secondary reconstructive procedures: timing of surgery, intraoperative findings, the method of brachial plexus reconstruction, motor assessment of relevant functions, and the percentage of a satisfactory outcome as per the criteria shown in Table 1.

**Table 1:** Postoperative motor assessment in children who underwent primary brachial plexus reconstruction

Function	Scoring or measurement of function	Definition of a satisfactory functional outcome
Shoulder abduction	Measured as degrees of shoulder abduction	Abduction 120^o^ or more
Shoulder external rotation	1 = the hand reaches the abdomen or thorax; 2 = the hand reaches the mouth 3 = the hand reaches the ear; 4 = the hand reaches the occiput; 5 = normal external rotation	A score of 3 or more
Elbow flexion and extension	0 = no motion, 1 = active motion with gravity eliminated, 2 = active motion against gravity, 3 = active motion against resistance reaching ≤½ normal range, 4 = active motion against resistance reaching >1/2 normal range, 5 = normal	A score of 4 or 5
Wrist extension	0 = non-functional (no active extension or extension only with gravity eliminated) 1 = active wrist extension to less than neutral 2 = active wrist extension to neutral or more than neutral 3 = normal wrist extension	A score of 2 or 3
Digital extension	0 = non-functional (no active extension or extension only with gravity eliminated) 1 = active digital extension to less than 1/2 range of motion 2 = active digital extension to more than ½ range of motion 3 = normal digital extension	A score of 2 or 3

Note that wrist flexion and hand function are not included in the assessment because C8-T1 roots are not affected in extended Erb’s palsy

## Results

Forty-six consecutive infants with unilateral extended Erb’s palsy and single root avulsion were included in the study. The time of surgery ranged from 3.5–6 months (mean of 4.9 months). Intraoperative findings showed rupture of the C5/C6 roots and avulsion of the C7 root in 44 cases. In the remaining two cases, the avulsed root was the C6 root. The method of brachial plexus reconstruction was the same in all patients. Three cable grafts were utilized to reconstruct the anterior division of the upper trunk (for biceps re-innervation). One cable graft was utilized to reconstruct the suprascapular nerve (for re-innervation of the supra-and infra-spinatus muscles). The remaining cable grafts were distributed to the posterior division of the upper trunk (the deltoid) and the middle trunk (extension of the elbow/wrist/digits). The number of cable grafts for the deltoid/extensors was not always specifically mentioned in the operative notes, but usually four cable grafts were equally distributed between the posterior division of the upper trunk and the middle trunk. The follow-up time for the current study (defined as final follow-up prior to any secondary procedures) ranged from 3 to 10 years (average 5.3 years) after surgery. The percentages of satisfactory outcomes at final follow-up are shown in Table 2. The outcome was satisfactory in all but one patient for elbow flexion and in all patients for elbow extension and digital extension. Most patients (84.8%) also had a satisfactory outcome for wrist extension. A less satisfactory outcome was noted for shoulder external rotation (65.2%) and shoulder abduction (56.5%).

**Table 2:** The percentage of satisfactory outcome at final follow-up in the study group (*n* = 46)

Function	% of patients with a satisfactory outcome^a^
Shoulder abduction	56.5
Shoulder external rotation	65.2
Elbow flexion	97.8
Elbow extension	100
Wrist extension	84.8
Digital extension	100

^a^The definition of a satisfactory outcome is shown in Table 1

## Discussion

In extended Erb’s palsy, the priority of reconstruction is always given to elbow flexion [1, 5]. Hence, three cable grafts were always used in the current series to reconstruct the anterior division of the upper trunk regardless of the length of the defect. This is translated in an excellent recovery of the biceps as shown in Table 2.

The second priority of reconstruction is usually given to the suprascapular nerve (shoulder external rotation). The two main options of neurotization of the suprascapular nerve are: nerve grafting from the available (ruptured) roots of the brachial plexus and spinal accessory nerve transfer. There are two studies in the literature comparing these two different methods of reconstruction in OBBP, and both studies documented no significant differences in the outcome [7, 8]. In our study, all patients underwent the nerve grafting method and 65.2% were considered to have a satisfactory outcome. This outcome is similar to other studies [7, 8]. It should be noted that this is a “functional” outcome documenting the ability of the child to reach the ear or the back of the head. Postsuprascapular nerve reconstruction, we noted (as well as others [8]) that many of the children reach their heads with a variable degree of a “trumpet” posture, using the help of shoulder abduction. Hence, this functional outcome should not be considered as a measure of true external rotation of the shoulder as emphasized by Pondaag et al. [8]. In our series, the distal nerve graft coaptation varied depending on the length of the scar within the trunks. In most cases, one cable graft was coapted to the isolated suprascapular nerve, and in these cases, the relatively poor true external rotation of the shoulder might have been related to either a scarred suprascapular nerve (i.e., inadequate nerve debridement) or due to a second injury site at the suprascapular notch [8]. In few cases, the scarred area within the trunk was proximal to the origin of the suprascapular nerve, and in these cases, the cable graft meant for suprascapular nerve reconstruction might have missed the exact topographic area of the nerve within the upper trunk. Furthermore, we do not do intraoperative biopsies of the proximal cut-ends of the roots. Hence, another possibility is that the proximal spot within the C5 root where the graft is connected might be scarred or sensory.

In the current study, a 100% satisfactory outcome was noted for elbow extension and digital extension. Such an outcome is probably related to the fact that the triceps and digital extensors are supplied by both the C7 and C8 roots. Since the C8 root is still functioning in patients with extended Erb’s palsy, it is hard to differentiate the contribution of nerve grafting versus the intact C8 root to this satisfactory outcome. In contrast, wrist extension is mostly from the C7 root. Our rate of satisfactory outcome for wrist extension was high (84.8%). Furthermore, the problem of lack of postoperative functional wrist extension in children with extended Erb’s palsy is easily solved by tendon transfers [9]. So, we were happy with the outcome of wrist extension.

The lowest rate of satisfactory outcome in the current study (see Table 2) was for shoulder abduction (only 56.5% of children were able to abduct the shoulder 120^o^ or more). The reason for this is not clear and may be multi-factorial. Many older children with OBBP develop secondary osseous deformity at and around the shoulder joint and this may affect shoulder abduction [10, 11]. Another factor may be related to non-compliance with physiotherapy exercises in older children which invites contractures of shoulder adductors/internal rotators. A third factor may be related to insufficient neurotization of the posterior division of the upper trunk. Finally, it is important to note that our definition of a satisfactory shoulder abduction is rather strict (120^o^ or more) compared to other authors [12] who consider the outcome satisfactory if the abduction is greater than 90^o^.

The main strength of the current study is the presentation of a specific group of infants with extended Erb’s palsy treated at one center using the same assessment methods and operated by one surgeon using the same surgical strategy. However, there are several weaknesses which are inherent in most studies on obstetric palsy. Firstly, the study was retrospective and intraoperative notes regarding the exact number of cable grafts was not always detailed. Secondly, the definition of a “satisfactory” outcome in all obstetric palsy centers (including ours) is arbitrary. For example, a satisfactory outcome of shoulder abduction may be defined as over 90^o^ or over 120^o^ as mentioned above. Another example is the functional assessment of shoulder external rotation elbow flexion which is scored independently from the involved muscles. Finally, a root avulsion is not only a lesion at the foramen but extends into the myelin, and the trauma will not always respect the “limits” or zones of one root. The visibly “intact” root adjacent to the ruptured or avulsed roots may be scarred. For example, in extended Erb’s palsy with C7 rupture, the C8 root may have some scarring. Also, in cases of C6 avulsion, both the C5 and C6 roots may be harmed at the foramen. We assess the root quality before grafting by examining the cut edge of the root by the microscope, and we do not send intraoperative biopsies for root quality assessment.

In conclusion, primary intraplexus neurotization of the brachial plexus in infants with extended Erb’s obstetric palsy and single root avulsion gives a satisfactory outcome at the elbow, as well as wrist, and digital extension. The lower satisfactory rate for shoulder external rotation is probably not related to the method of reconstruction of the suprascapular nerve. The lowest rate of a satisfactory functional outcome was for shoulder abduction, and the reason is probably multi-factorial. However, one may argue that an extra cable nerve graft may have been available for neurotization of the posterior division of the upper trunk if the suprascapular nerve was neurotized from the spinal accessory nerve in the current series. This would require a comparison in another study.
